# Comprehensive Analysis of Phytochemical Compounds in Seeds of Indigenous Grape Varieties Cultivated in Southeastern Anatolia: Fatty Acids, Tocopherols, Phytosterols, Vitamins, and Antioxidant Properties

**DOI:** 10.3390/molecules31183168

**Published:** 2026-09-09

**Authors:** Mehmet İlhan Odabaşioğlu, Atilla Çakır, Nesrin Karaca Sanyürek, Fırat İşlek

**Affiliations:** 1 Department of Horticulture, Faculty of Agriculture, Adıyaman University, Kâhta 02400, Türkiye; milhanodabasioglu@gmail.com; 2 Department of Horticulture, Faculty of Agriculture, Bingöl University, Bingöl 12000, Türkiye; 3 Department of Food Engineering, Faculty of Engineering, Munzur University, Tunceli 62000, Türkiye; nkaraca@munzur.edu.tr; 4 Department of Plant and Production Technologies, Faculty of Applied Sciences, Muş Alparslan University, Muş 49250, Türkiye; firatislek12@gmail.com

**Keywords:** antioxidant capacity, DPPH, fatty acids, MDA, grape seeds, ripening stage, skin colour, tocopherols, phytosterols, *Vitis vinifera* L.

## Abstract

Aim: In this study, the phytochemical composition of the seeds of twelve different local grape (*Vitis vinifera* L.) varieties traditionally cultivated in the Southeastern Anatolia Region (Diyarbakır, Türkiye) was investigated using a holistic approach. The main objective of the study was to evaluate the associations of the effects of berry skin colour, the ripening stage and utilization type on the fatty acid profile, tocopherol and phytosterol contents, vitamin K and D levels, antioxidant capacity (DPPH) and lipid peroxidation (MDA) of grape seeds. Method: The seeds of grape varieties belonging to four different colour groups, grown on their own roots, were harvested during the 2017 growing season and analysed. Fatty acid composition was determined by gas chromatography (GC), vitamin and phytosterol contents by HPLC-UV, MDA content by the thiobarbituric acid reactive substance (TBARS) method, and antioxidant capacity by the DPPH radical scavenging assay. Findings: Linoleic acid was identified as the predominant fatty acid in all varieties (53.68–67.06%), followed by oleic acid (13.80–27.32%). α-Tocopherol was identified as the predominant tocopherol form (1.597–4.703 mg kg^−1^), whilst β-sitosterol was the phytosterol component present at the highest level (915.67–3340.42 µg g^−1^). Statistically significant varietal differences were detected for most measured phytochemical parameters. Group-wise differences associated with berry skin colour and ripening stage were observed for selected traits. Overall, varietal differences appeared to be more pronounced than those associated with berry skin colour or ripening stage. It was observed that the differences in phytochemical composition between the various commercial classifications of grapes were very limited. Result: The findings reveal that the seeds of the local grape varieties studied exhibit a rich phytochemical profile and represent an important source of bioactive compounds that could be utilised, particularly in the functional food, nutraceutical and cosmetics industries. The ‘Tahannebi’ variety stood out as the one with the highest values across many parameters. Differences observed among samples of the same cultivar collected from different vineyards suggest that vineyard-specific conditions may contribute to variation in selected phytochemical traits. Future multi-year and multi-location studies are needed to confirm the stability of these phytochemical profiles and to further clarify genotype × environment interactions.

## 1. Introduction

The grape (*Vitis vinifera* L.) is one of the fruit species with the largest cultivation area worldwide and has held a central position in human dietary culture throughout history. According to data from the Food and Agriculture Organisation of the United Nations (FAO), global grape production exceeds approximately 78 million tonnes annually, with a significant proportion of this production being used to produce processed products such as wine, raisins, fruit juice and vinegar [1]. By-products arising from grape processing include components such as skins, seeds and stems, which account for approximately 20% of the total fruit weight [2]. According to [3], the quantity of grape seeds generated as waste by the food industry amounts to approximately 1.4 million tonnes annually. These components, which were long regarded as waste, have in recent years become the focus of scientific research due to the bioactive compounds they contain.

Among grape-processing by-products, grape seeds are particularly noteworthy because, although they account for only approximately 3–6% of the fruit’s total weight, they possess an extremely rich biochemical composition, including phenolic compounds, fatty acids, tocopherols, phytosterols, and various vitamins [4,5]. The positive effects of these compounds on human health are evident across a wide range of areas, from cardiovascular protection, anti-inflammatory and anti-carcinogenic activities to the support of bone health [5,6]. For this reason, grape seeds are attracting increasing interest in the fields of functional foods, nutraceuticals, and pharmaceutical applications.

Within this diverse biochemical matrix, the lipid fraction of grape seeds is particularly notable for its high content of unsaturated fatty acids (UFAs). Linoleic acid is the primary fatty acid in grape seed oil and constitutes a large proportion of its lipid composition [7]. The cholesterol-lowering effect of this fatty acid and its potential to reduce the risk of cardiovascular disease have been supported by epidemiological and clinical studies [8]. Furthermore, other fatty acids such as oleic acid, palmitic acid and stearic acid also constitute important components of the grape seed lipid profile. Research in the literature has shown that between 5 and 34 different fatty acids can be found in grape seeds, and that their composition may vary depending on genotype and environmental factors [9,10,11].

Beyond fatty acids, grape seeds also contain important lipid-soluble bioactive compounds, particularly tocopherols and phytosterols. Tocopherols are potent fat-soluble antioxidants; in particular, the α-tocopherol (vitamin E) form plays a critical role in protecting biological membranes against oxidative damage [12]. It has been reported that α-tocopherol is the predominant form in grape seed oil, whilst γ-tocopherol is also present at significant levels [13,14]. Phytosterols, on the other hand, are plant-derived sterol compounds that have the capacity to lower plasma LDL-cholesterol levels by reducing cholesterol absorption [15,16,17]. β-sitosterol is the most abundant phytosterol in grape seeds, whilst stigmasterol and campesterol are also among the significant components [18,19].

In addition to compositional characterization, evaluating antioxidant capacity and oxidative damage is essential for understanding the functional properties of grape seeds. The DPPH (2,2-diphenyl-1-picrylhydrazyl) radical scavenging assay, frequently used to assess antioxidant capacity, is recognised as a reliable method for determining the free radical scavenging potential of grape seed extracts [20,21,22,23]. On the other hand, malondialdehyde (MDA), an end product of lipid peroxidation, is widely used in biochemical research as an indirect indicator of oxidative stress at the cellular level [24,25]. The simultaneous assessment of these two parameters is of great importance for understanding the dynamic balance between oxidative damage and antioxidant defence systems.

Recent research has shown that, in addition to being rich in phenolic compounds and lipids, grape seeds also contain trace amounts of vitamins K and D. However, the levels of these compounds can vary significantly depending on numerous factors, such as variety, ecology, harvest time, skin colour and altitude [19,26].

Given that the levels of these phytochemical constituents may vary markedly among genotypes and growing environments, their characterization is particularly relevant in major centres of grape biodiversity. Türkiye is one of the world’s most important centres of grape biodiversity, and the Southeastern Anatolia Region in particular, with its millennia-old viticultural tradition, is home to a large number of local grape varieties. The systematic characterisation of the phytochemical profiles of the seeds of these local varieties is of strategic importance both for the conservation of genetic resources and for the evaluation of the industrial potential of these resources.

Whilst there are numerous studies in the existing literature that examine different aspects of grape seed composition separately, studies that comprehensively evaluate parameters such as fatty acids, tocopherols, phytosterols, vitamins, antioxidant capacity (DPPH) and lipid peroxidation (MDA) within the same research framework are quite limited. Furthermore, statistically elucidating the interrelationships between these components will make a significant contribution to a comprehensive understanding of the functional properties of grape seeds.

In this context, the aim of the present study is to determine the fatty acid composition, tocopherol and phytosterol contents, levels of vitamins K and D, antioxidant activity (DPPH) and lipid peroxidation (MDA) parameters in seed samples from different local grape varieties grown in the Southeastern Anatolia Region; to statistically analyse the relationships between these components and to elucidate the associations of berry skin colour, ripening stage, and utilization type with the aforementioned parameters.

## 2. Results and Discussion

### 2.1. Vitamin Composition (Vitamins K and D)

When the vitamin K and D content of grape seed samples was examined, statistically significant differences at a high level were detected between varieties for all vitamin components (*p* < 0.01). This indicates that vitamin accumulation is strongly dependent on genotypic structure (Table 1). However, when grape varieties were grouped according to the evaluation methods used, no statistically significant differences were found in the K and D vitamin content of the seeds between the evaluation method groups.

A wide variation in vitamin K_1_ content was observed among the varieties, ranging from 0.063 to 0.833 µg g^−1^. The ‘Şitu’ variety had the highest Vitamin K_1_ value at 0.833 µg g^−1^, followed by the ‘Tahannebi’ (0.653 µg g^−1^) and ‘Çirbeyt (*V2*)’ (0.533 µg g^−1^) varieties. The lowest K_1_ level was determined in the ‘Habo’ variety (0.063 µg g^−1^). Vitamin K_2_ was generally found at low levels, with seven varieties having K_2_ levels below the detection limit. The ‘Hatun Parmağı’ variety stood out as having the highest K_2_ content at 0.387 µg g^−1^ (*p* < 0.01).

A considerable variation in vitamin D_2_ levels was observed among the varieties. The ‘Asuri’ variety had the statistically highest D_2_ level at 1.603 µg g^−1^, whilst vitamin D_2_ could not be detected in four varieties. As for vitamin D_3_, the highest value was recorded in the ‘Çirbeyt (*V2*)’ variety at 0.577 µg g^−1^; however, this vitamin was not detected in samples taken from the *V3* location of the same variety.

**Figure 1:**
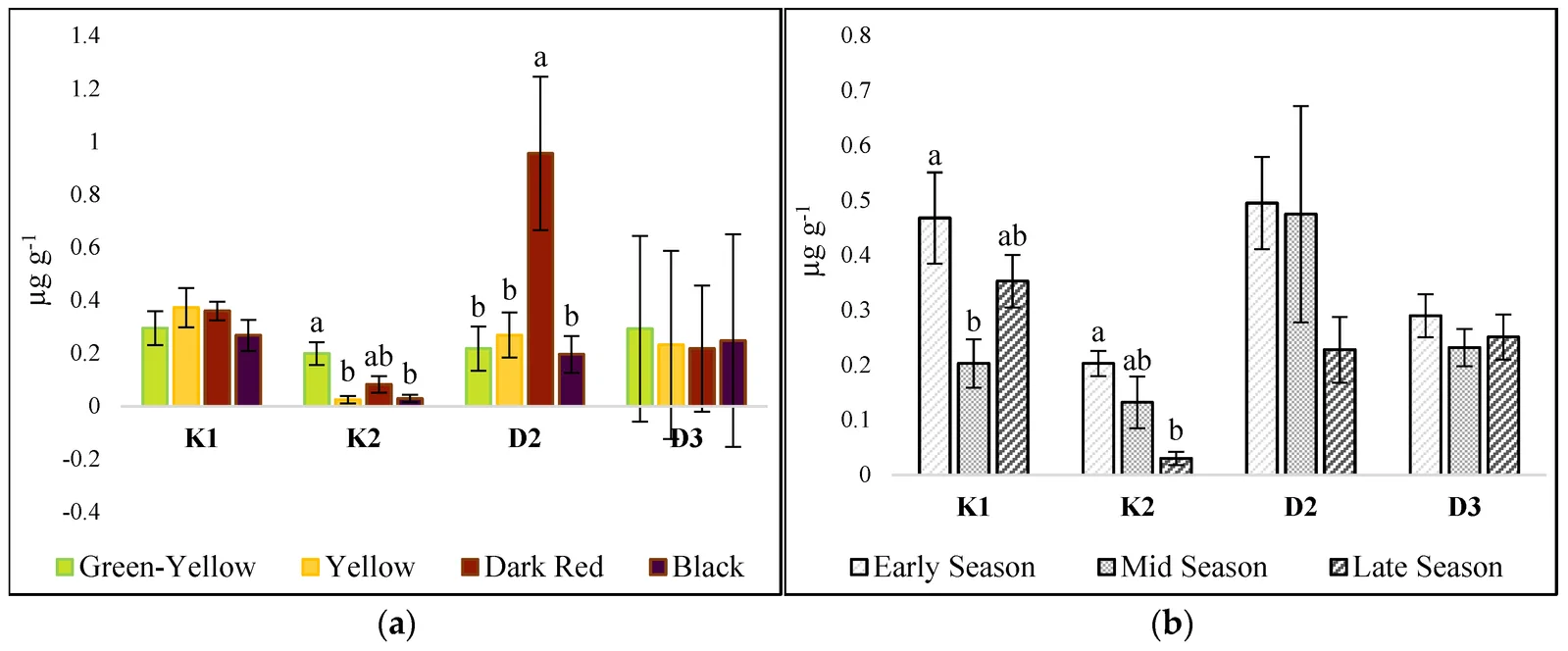
Variation in vitamin K and D content in grape seeds across groups based on berry skin colour (**a**) and ripening period (**b**). Different lowercase letters indicate statistically significant differences among groups according to Tukey’s multiple comparison test.

When the grape varieties examined in this study were grouped according to their berry skin colour, statistically significant differences were found between the berry skin colour groups in terms of vitamin K_2_ and D_2_ content (Figure 1a). The Green-Yellow skin colour group was found to have a higher vitamin K_2_ content (0.199 µg g^−1^), whilst the Dark Red group had a higher vitamin D_2_ content (0.955 µg g^−1^) compared to the others. In contrast, the skin colour groups were found to be similar in terms of vitamin K_1_ and D_3_ content. Furthermore, the groups classified by fruit ripening stage showed statistically significant differences in K_1_ and K_2_ vitamin content in the kernels; however, no significant difference was detected between the groups in terms of D_2_ and D_3_ vitamin content (Figure 1b). The vitamin K content in the seeds of early-maturing varieties (K_1_ 0.468 µg g^−1^ and K_2_ 0.203 µg g^−1^, respectively) was found to be higher than that of mid-season and late-season varieties.

The significant differences (*p* < 0.01) observed among varieties in vitamins K and D suggest genotype-associated variation in vitamin accumulation. However, environmental and growing conditions may also contribute to these differences. Indeed, similar findings were reported by Akbaba et al. [26] and Çakır et al. [19] in the grape varieties they studied. In particular, the higher levels of vitamin D_2_ found in dark-coloured varieties can be explained by the relationship between the pigmentation characteristics of the tissues and the UV-induced photochemical conversion of ergosterol. Ergosterol is a key sterol precursor that can be converted into vitamin D_2_ when exposed to ultraviolet radiation. It has been reported that differences in the optical properties of the tissue surface, pigment density and UV penetration capacity may influence the efficiency of this conversion [27,28].

The fact that early-maturing varieties exhibit higher levels of vitamins K_1_ and K_2_ can be explained by the photosynthetic mechanism exhibiting more intense metabolic activity during the short growing season and the close relationship between vitamin K biosynthesis and chloroplast metabolism. Vitamin K_1_ (phylloquinone) is a cofactor involved in the photosynthetic electron transport chain and therefore accumulates in tissues with high photosynthetic activity [29]. On the other hand, the accumulation of vitamin K_1_ in plant tissues may vary depending on the level of light exposure [30].

### 2.2. Tocopherol Composition

Statistically significant differences were observed between grape varieties in terms of the tocopherol content of the seeds (*p* < 0.01). α-Tocopherol was identified as the predominant component in all grape varieties examined (Table 2). The α-tocopherol content in grape seeds ranged from 1.597 to 4.703 mg kg^−1^, with the ‘Şitu’ and ‘Tahannebi’ varieties identified as having the highest α-tocopherol content. In the seeds of the ‘Siyahi’ variety, however, this component was found in lower quantities than in the other varieties examined. The δ-tocopherol content ranged from 0.030 to 1.027 mg kg^−1^, with the ‘Çirbeyt (*V2*)’ variety having the highest content and the ‘Boğazkere’ variety having the lowest. Furthermore, differences in δ-tocopherol content were observed between samples (*V1*, *V2*, *V3*) taken from different vines of the ‘Çirbeyt’ variety.

**Figure 2:**
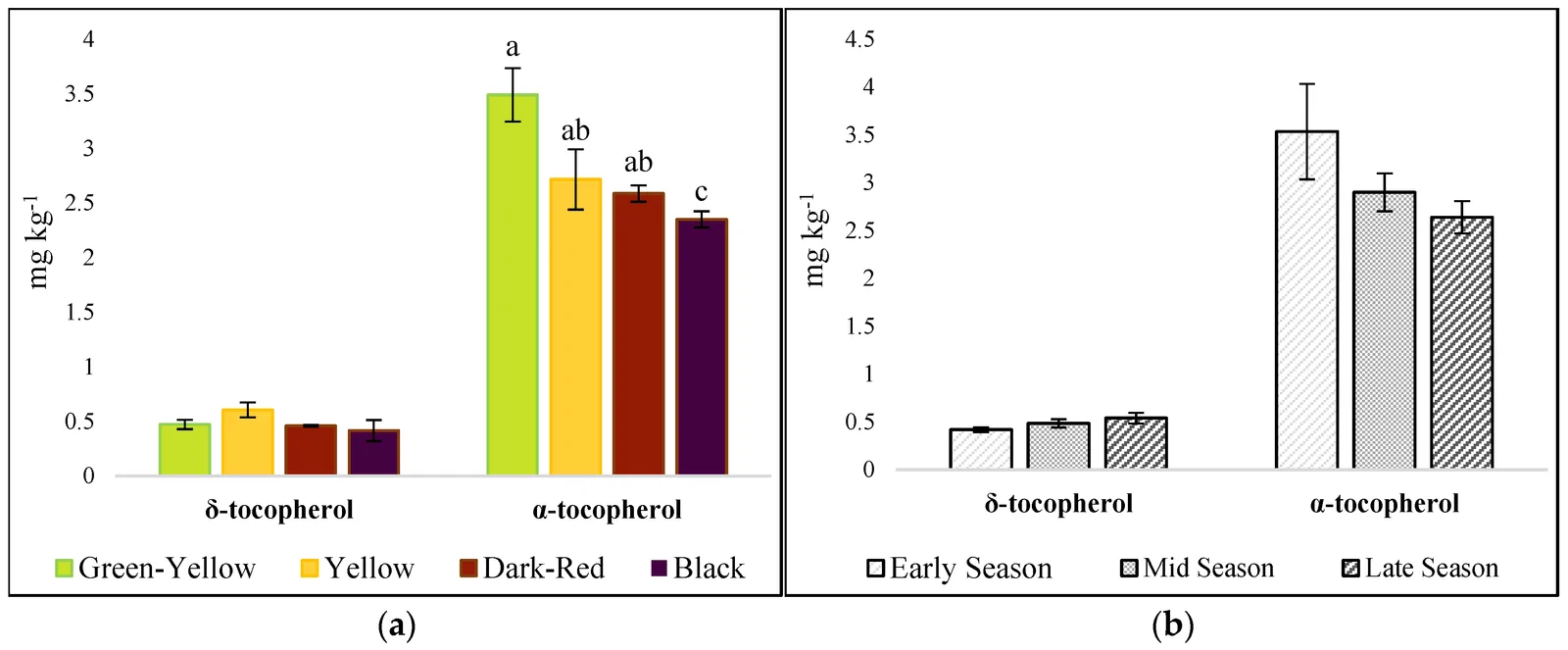
Variation in tocopherol content in grape seeds across berry skin colour (**a**) and ripening stage (**b**) groups. Different lowercase letters indicate statistically significant differences among groups according to Tukey’s multiple comparison test.

In the evaluation based on husk colour groups, it was determined that the α-tocopherol content in grape seeds decreased as the husk colour darkened along a colour scale ranging from green-yellow to black (*p* < 0.05). In contrast, the δ-tocopherol content in the seeds did not show any statistically significant differences between the berry skin colour groups (*p* > 0.05) (Figure 2a). Vujasinovic et al. [31] and Carmona-Jimenez et al. [14], who reached similar findings, reported that white grape varieties contain higher amounts of α-tocopherol in their seeds compared to red grape varieties. The effects of the ripening period (Figure 2b) and evaluation method (Table 2) groups on the content of both tocopherol compounds in grape seeds were not found to be statistically significant (*p* > 0.05).

The identification of α-tocopherol as the predominant tocopherol form in all varieties once again confirms the potential of grape seeds as a natural source of vitamin E. Whilst Crews et al. [32] reported α-isomers as the dominant component in grape seed oils of different origins, Shinagawa et al. [13] emphasised that this component makes a significant contribution to the oxidative stability of grape seed oil. The α-tocopherol content values we determined in the varieties examined in our study are similar to those reported by Carmona-Jimenez et al. [14] for varieties grown in southern Spain.

The detection of higher α-tocopherol levels in green-yellow skinned varieties stands out as a notable biochemical finding. The higher α-tocopherol levels observed in light-coloured varieties may be associated with differences in metabolic allocation between tocopherol- and pigment-related pathways [33,34]. However, the underlying metabolic mechanism was not directly investigated in the present study and therefore requires further targeted analysis.

### 2.3. Phytosterol Composition

Significant statistical differences were observed between the varieties in terms of phytosterol components (β-sitosterol, stigmasterol and ergosterol) (*p* < 0.01). β-sitosterol was identified as the predominant phytosterol component in all varieties. Among the grape varieties examined, the ‘Tahannebi’ variety had the highest β-sitosterol and stigmasterol content in its seeds. In contrast, the highest ergosterol content was found in the seeds of the ‘Boğazkere’ variety. The ‘Mazrone’ variety, on the other hand, was identified as having the lowest phytosterol content in the seeds amongst the grape varieties examined (Table 3). Another notable finding in our study was that the phytosterol content varied in samples of the ‘Çirbeyt’ variety taken from different vineyards. Samples taken from location *V1* were found to have higher β-sitosterol and ergosterol content, whilst those from location *V2* had higher stigmasterol content.

Although the average stigmasterol and β-sitosterol content in the seeds of table grape varieties, and the average ergosterol content in the seeds of wine grape varieties, were found to be higher than those of the others, no statistically significant difference was detected between the grape evaluation groups in terms of the amount of phytosterol components (*p* > 0.05).

**Figure 3:**
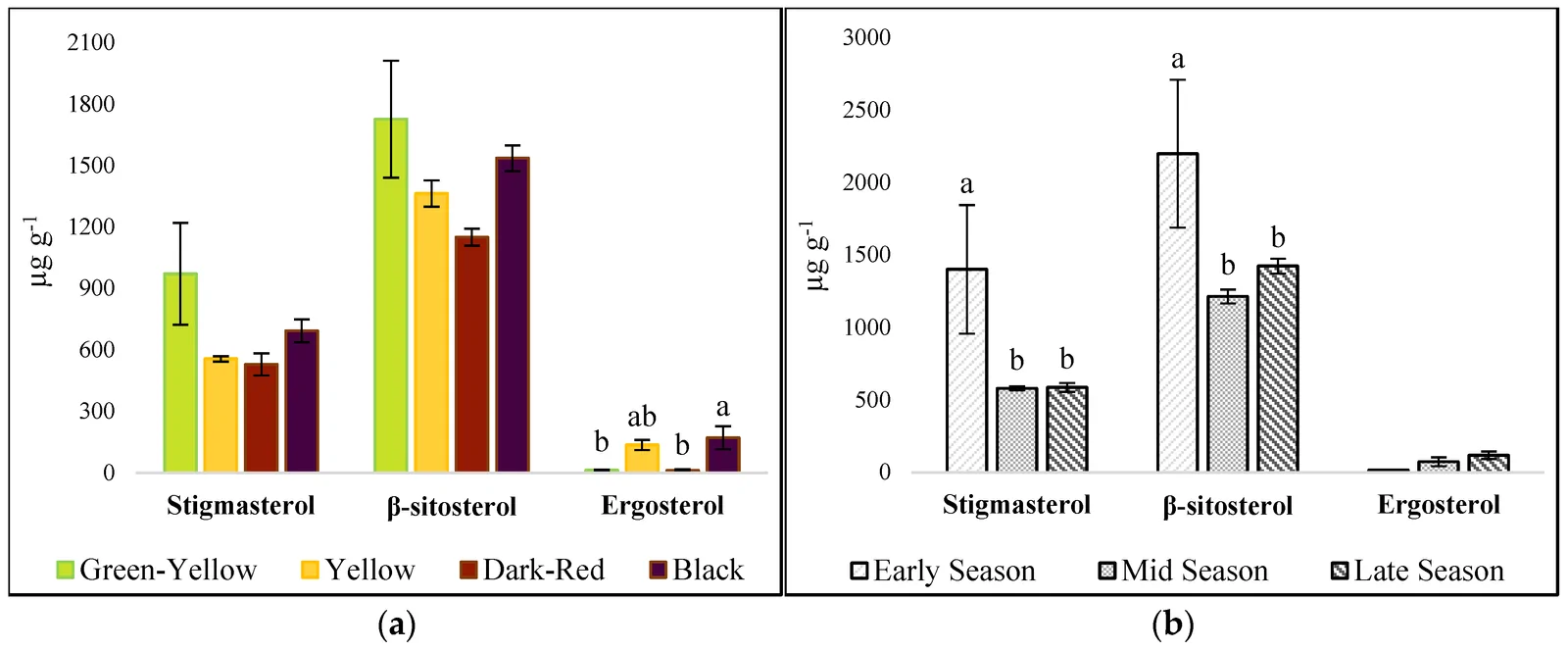
Variation in phytosterols in grape seeds across berry skin colour (**a**) and ripening stage (**b**) groups. Different lowercase letters indicate statistically significant differences among groups according to Tukey’s multiple comparison test.

It was found that the grape seed skin colour groups showed statistically significant differences (*p* < 0.01) in terms of ergosterol content, and that this compound was found to be higher in varieties with a ‘Black’ skin colour (Figure 3a). It was found that the stigmasterol and β-sitosterol content in the seeds was not affected by berry skin colour (*p* > 0.05); however, statistically significant differences (*p* < 0.01) were observed between the berry ripening period groups. The levels of both stigmasterol and β-sitosterol in the kernels of early-maturing varieties were found to be higher than those in mid-season and late-season varieties (Figure 3b).

The identification of β-sitosterol as the predominant phytosterol component is fully consistent with previous studies conducted on various vegetable oils [15,35] and different grape varieties [36]. On the other hand, Piironen et al. [16] emphasised that stigmasterol is one of the most abundant sterol group compounds in commonly consumed vegetable oils. Previous researchers have demonstrated that phytosterol compounds present in grape seeds may vary between *Vitis* species, *Vitis vinifera* L. varieties and even *Vitis* sp. hybrids [37,38,39]. In this respect, our findings are consistent with those of previous researchers. Some studies have reported that phytosterols in grape seeds may be influenced by ecological conditions and cultural practices carried out in vineyards [40,41,42]. The variation in phytosterol content observed in samples of the Çirbeyt variety collected from different vineyards may be attributable to differences in the cultural practices applied in the vineyards.

The ability of phytosterols to lower plasma LDL-cholesterol levels by reducing cholesterol absorption has been strongly supported by epidemiological studies [17]. Furthermore, it has been reported that β-sitosterol offers protection against candidiasis, whilst stigmasterol inhibits cholesterol synthesis in the liver and intestinal absorption [43]. In this context, the high β-sitosterol (3340.42 µg g^−1^) and stigmasterol (2394.58 µg g^−1^) contents of the ‘Tahannebi’ variety in particular suggest that this variety could be prioritised for consideration in nutraceutical and functional food applications.

The fact that the stigmasterol and β-sitosterol contents of early-ripening varieties were found to be significantly higher than those of other groups indicates that the phenological development process has a decisive influence on phytosterol accumulation. Indeed, Rubio et al. [44] found that, in parallel with berry development, the β-sitosterol content in grape seeds decreased, whilst the stigmasterol content increased. Ruggiero et al. [40], on the other hand, reported that phytosterols other than stigmasterol in the seeds decreased significantly after berry drop compared to the period before berry drop. This finding can be interpreted as suggesting that a shorter growing season in early-maturing varieties may increase metabolic concentration. The higher ergosterol levels in black-coloured varieties, meanwhile, indicate the existence of complex interactions between pigmentation and sterol metabolism.

### 2.4. Lipid Peroxidation (MDA) and Antioxidant Activity (DPPH)

The grape varieties examined in this study exhibited statistically significant differences (*p* < 0.01) in terms of MDA content and antioxidant capacity (DPPH) in their seeds. MDA content ranged from 34.51 to 314.50 nmol g^−1^, with the ‘Tahannebi’ variety identified as having the highest level of lipid peroxidation. The lowest MDA values were recorded in the ‘Hasani’ (34.51 nmol g^−1^) and ‘Habo’ (35.50 nmol g^−1^) varieties (Table 4).

DPPH radical scavenging activity varied within the range of 91.41–96.89%. Whilst the ‘Tahannebi’ variety exhibited the highest antioxidant activity, the lowest antioxidant activity was observed in samples taken from the *V3* location of the ‘Çirbeyt’ variety. The fact that differences were observed between samples taken from different vines of the same variety, both in terms of lipid peroxidation and antioxidant capacity, indicates that the physiological response mechanism exhibited by varieties of the species *Vitis vinifera* L. to environmental factors is highly dynamic and flexible. Indeed, Akbaba et al. [42] also found that MDA and DPPH values in the seeds of the ‘Kohnu’ grape variety, grown in different regions, differed significantly.

**Figure 4:**
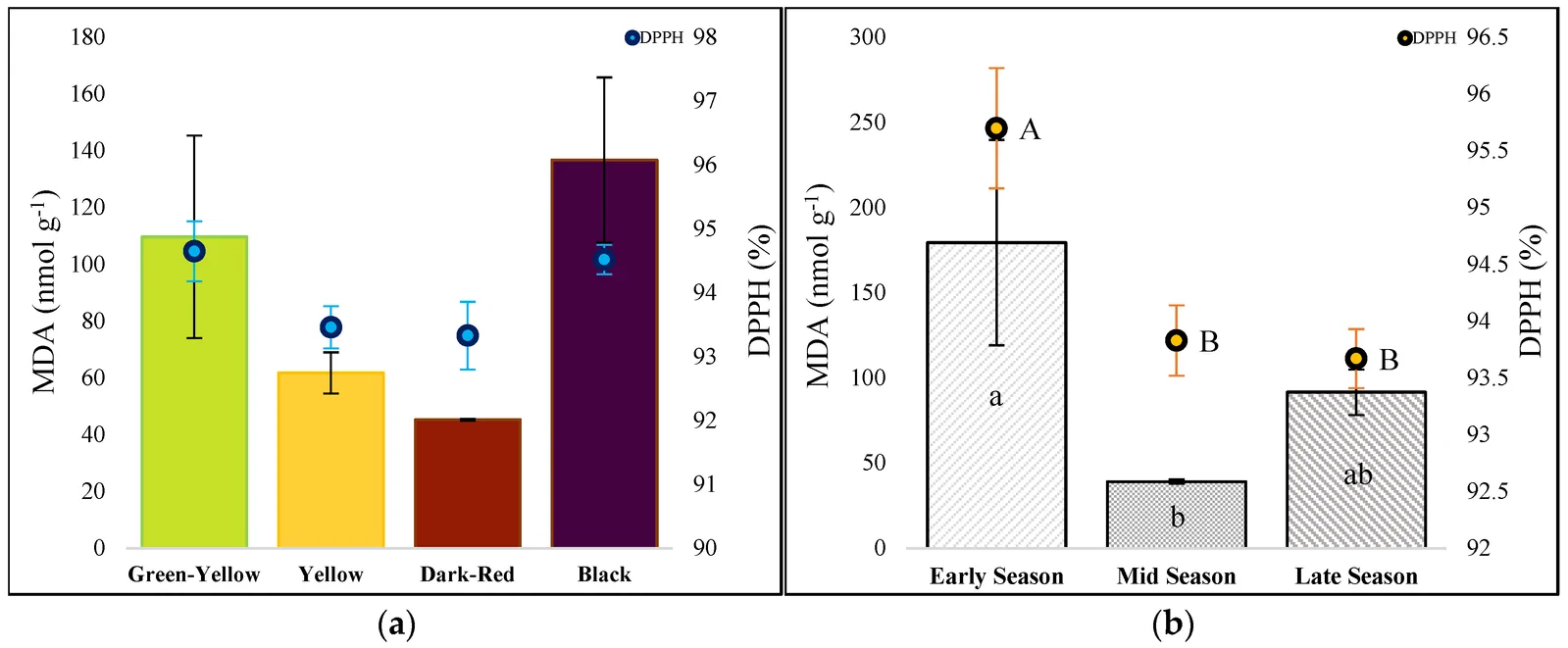
Variation in MDA content and antioxidant capacity (DPPH) in grape seeds across berry skin colour (**a**) and ripening stage (**b**) groups. Different lowercase letters indicate statistically significant differences among groups according to Tukey’s multiple comparison test.

In our study, the fact that varieties with high MDA values simultaneously exhibited high DPPH activity indicates the presence of a positive correlation (r = 0.55, *p* < 0.01) between oxidative stress and antioxidant defence mechanisms. When grape varieties were grouped according to evaluation methods (Table 4) and skin colour (Figure 4a), no statistically significant differences were found between the groups in terms of seed MDA content and antioxidant capacity (*p* > 0.05). Katalinic et al. [45] highlighted that the free radical scavenging capacities of grape varieties may be related to their polyphenolic content. In numerous studies examining grape skin or pulp + skin tissues, findings are frequently reported in the literature indicating that varieties with coloured skins (ranging from red to black) possess higher antioxidant capacity compared to those with green or green-yellow skins [46,47,48]. On the other hand, Niu et al. [49] reported that coloured varieties do not always outperform white varieties in terms of antioxidant capacity. Çakır et al. [19], however, reported that lipid peroxidation and antioxidant capacity in grape seeds did not vary according to berry skin colour groups, and that the main factor influencing these parameters was genotype. Furthermore, findings in the literature indicating that antioxidant and anti-radical capacities exhibit significant variations even among clones of a single grape variety [50,51] constitute important evidence of the evolutionary plasticity of the genus *Vitis*.

In the grouping based on the ripening stage of the berries, it was found that early-ripening varieties had higher values for both MDA (179.50 nmol g^−1^) and DPPH (95.70%) compared to the other groups (*p* < 0.01) (Figure 4b). The parallel trend observed at the variety level between the MDA and DPPH parameters strongly suggests that oxidative stress and antioxidant defence mechanisms are activated simultaneously in grape seed biochemistry. This finding can be interpreted within the framework of the concept of ‘oxidative adaptation’. It is known that in tissues with high metabolic activity, the synthesis of antioxidant enzyme systems and non-enzymatic antioxidants is increased in response to rising oxidative stress [52,53]. Rockenbach et al. [54] reported that grape seed extracts possess potent antioxidant activity, whilst also emphasising that this activity exhibits significant variations depending on the variety. Similarly, previous studies have reported that the antioxidant profile in the seeds, berry skins and raceme stalks of different grape varieties reflects metabolomic variation [55,56,57]. The fact that the ‘Tahannebi’ variety possesses both the highest MDA and the highest DPPH values indicates that oxidative metabolism is more active in this variety and, in parallel, that antioxidant defence mechanisms are induced more strongly.

The fact that both MDA and DPPH values in early-ripening varieties were found to be significantly higher than in other groups can be interpreted as suggesting that a short growing season may increase metabolic intensity, thereby enhancing both oxidative stress and the antioxidant response. This finding demonstrates that the phenological development process plays a decisive role not only at the morphological level but also at the biochemical level.

### 2.5. Fatty Acid Composition

An analysis of the fatty acid profiles in the seeds of grape varieties revealed statistically significant differences (*p* < 0.01) between grape varieties in terms of major fatty acid content (Table 5). However, it was determined that linoleic acid (C18:2, n-6) was the predominant fatty acid in all grape varieties. Linoleic acid was followed, in order, by oleic acid, palmitic acid and stearic acid. The same order has also been reported by previous researchers who examined different grape varieties [37,58]. In seed oils, linoleic acid ranged from 53.68% to 67.06%, with the ‘Mazrone’ and ‘Tahannebi’ varieties identified as having the highest linoleic acid content. In contrast, the ‘Hatun Parmağı’ variety had the highest oleic acid content (27.32%), the ‘Şitu’ variety had the highest palmitic acid content (10.10%), and the ‘Boğazkere’ variety had the highest stearic acid content (6.28%). The marked differences in linoleic and oleic acid levels among varieties are consistent with genotype-associated variation in fatty acid composition. The strong inverse relationship observed between linoleic and oleic acids (r = −0.97, *p* < 0.01) may be related to differences in desaturase-mediated lipid metabolism reported in grapevine [59]; however, desaturase enzyme activity or gene expression was not directly measured in the present study. The fact that linoleic acid and oleic acid have been identified as the predominant fatty acids in all varieties confirms that grape seed oil is a rich source of essential fatty acids. Indeed, in other vegetable oils commonly consumed as part of the daily human diet (such as rapeseed oil, maize oil, sunflower oil, soya bean oil and olive oil), the levels of linoleic acid and oleic acid are also considerably higher than those of other fatty acids [60]. Our findings regarding linoleic and oleic acid content are largely consistent with the results of previous studies conducted on varieties of the *Vitis vinifera* L. species in different geographical regions. Indeed, previous researchers have reported that in *Vitis vinifera* L. varieties grown in Türkiye, the linoleic acid content ranged from 51.6% to 70.1%, whilst the oleic acid content ranged from 15.8% to 31.6% [3,19,61]. The levels of these fatty acids in different grape (*V. vinifera* L.) varieties were reported by Beveridge et al. [62] in Canada as ranging from 66.7–73.6% and 12.6–18.9%, respectively; by Fernandes et al. [58] in Portugal in the ranges of 63.0–73.1% and 13.7–20.8%, and Lachman et al. [63] in the Czech Republic in the ranges of 69.2–77.2% and 9.9–18.0%. Furthermore, similar findings have been reported in different *Vitis* species, interspecific hybrids and vine rootstocks [64,65,66,67]. These findings are consistent with Matthäus’s [8] assessment that linoleic acid is the characteristic fatty acid of grape seed oil.

In studies comparing grape varieties based on the fatty acid composition of their seeds, it has been reported that the fatty acid composition of the seeds can vary depending on factors such as genotype, berry skin colour, berry ripeness, climatic variations, altitude, rootstocks, ecology, irrigation regime, etc. [3,11,32,44,63,68,69,70]. However, genotype is the most dominant of these factors. Indeed, in samples of the Çirbeyt variety taken from different vineyards, the fatty acid composition exhibited a more stable profile compared to the other phytochemicals we examined. This finding is consistent with the observation by Odabaşioğlu and Gürsöz [3] that, in the composition of grape seeds, genotype is a more dominant determining factor than the growing season and rootstocks. On the other hand, in the commercial production of grape seed oil, there are other factors that may influence oil content and/or fatty acid composition. These include the method of drying the grapes/seeds after harvest, the storage conditions of the seeds, and the oil extraction method [36,62,64,71,72].

When the varieties were grouped according to their commercial uses, the differences in fatty acid composition between the groups were limited to palmitic acid and myristic acid (Table 5 and Table 6). Baydar and Akkurt [73], who reached similar findings, reported that there was no clear difference in fatty acid composition between the wine and table grape varieties they examined.

**Figure 5:**
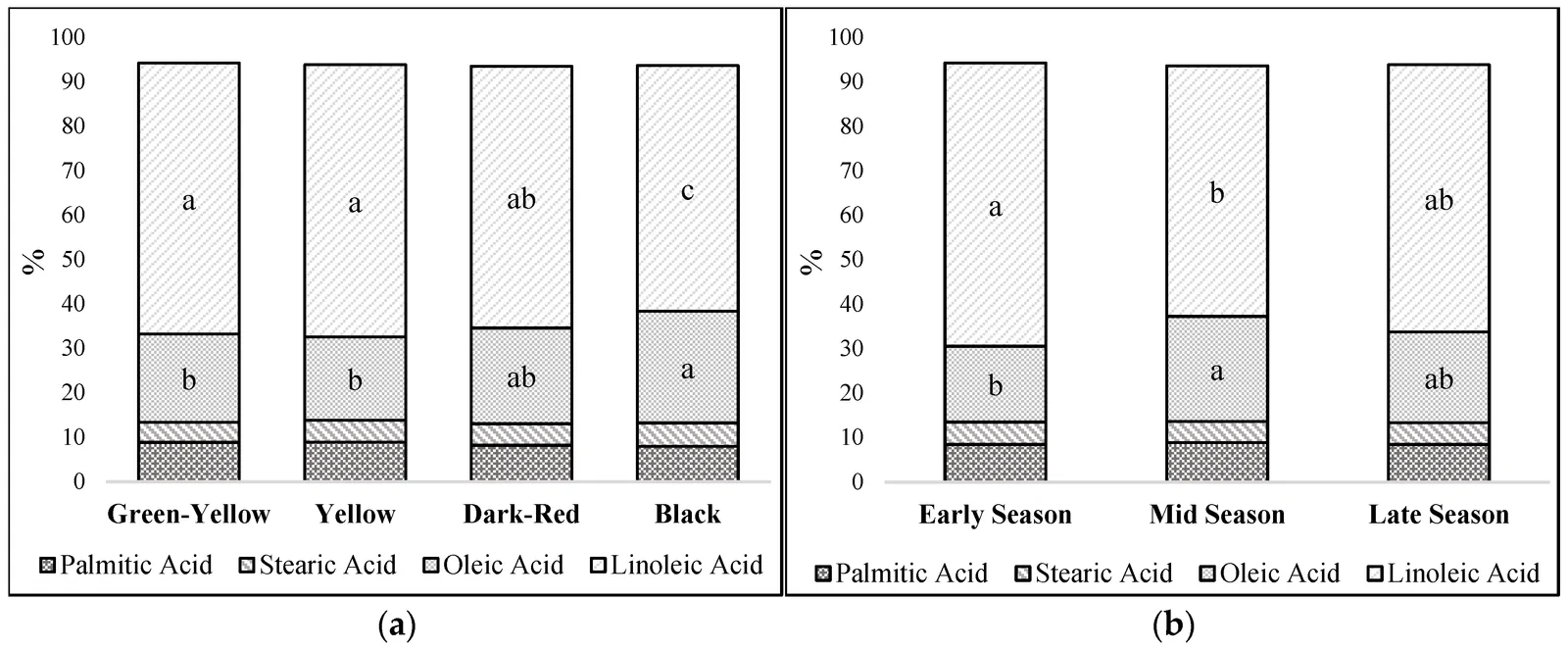
Variation in the major fatty acid composition of grape seeds across berry skin colour (**a**) and ripening period (**b**) groups. Different lowercase letters indicate statistically significant differences among groups according to Tukey’s multiple comparison test.

When varieties were classified according to skin colour, the groups with the highest linoleic acid content in their seeds were the Green-Yellow (60.97%) and Yellow (61.23%) skin colour groups, and these two groups were statistically distinct from the other colour groups (*p* < 0.01). In contrast, the oleic acid content of the Black husk colour group (25.15%) was found to be significantly higher than that of the other colour groups (*p* < 0.05). No statistically significant difference was detected between the peel colour groups in terms of palmitic acid and stearic acid content in the kernels (Figure 5a). The finding that the oleic acid ratio was higher and the linoleic acid ratio lower in the black-peel-coloured varieties is a particularly striking result. This phenomenon suggests that there may be a metabolic link between anthocyanin biosynthesis and lipid metabolism via a common precursor pool. Indeed, malonyl-CoA serves not only as a key precursor for fatty acid synthesis and elongation but also as a building block in flavonoid biosynthesis; furthermore, it has been reported that genetic interventions affecting this precursor pool alter both anthocyanin accumulation and metabolites associated with fatty acids and cuticular wax in some plant systems [74,75]. Meanwhile, similar findings regarding variations in oleic acid content in relation to skin colour have also been reported by Çakır et al. [19] in indigenous grape varieties cultivated in Southeastern Anatolia. However, some researchers argue that similar changes occur in the palmitic and stearic acid contents [11,76]. The differences between these studies may be due to variations in the grape varieties examined or the ecological conditions under which they were cultivated.

When the ripening stage groups were compared in terms of the fatty acid composition of the seeds, it was observed that linoleic acid and myristic acid were higher in the seeds of early-ripening varieties, whilst oleic acid, palmitoleic acid and trans-elaidic acid were higher in the seeds of mid- and late-season varieties (Figure 5b and Table 6). However, the other fatty acids present in the seeds were not affected by the ripening stages of the grape varieties (*p* > 0.05). According to Odabaşioğlu [11], the composition of major and minor fatty acids in grape seeds is influenced by the ripening period of the varieties; whilst linoleic acid, palmitic acid and stearic acid were found to be higher in early-ripening varieties, oleic acid was present in higher amounts in varieties ripening in the mid and late season. Çakır et al. [19], however, reported that linoleic acid was not affected by the ripening period, but that the oleic acid content was low in the seeds of early-ripening varieties. Taking into account the findings obtained and reports in the literature, it appears more appropriate to use mid- and late-season varieties with a high oleic acid content for the commercial production of grape seed oil, in order to maintain oxidative stability over the long term.

When the fatty acid composition of grape varieties was assessed according to the degree of saturation of the fatty acids, it was found that polyunsaturated fatty acids (PUFAs) were the predominant group in all varieties. Statistically significant differences (*p* < 0.01) were found between varieties in terms of PUFA, MUFA (monounsaturated fatty acids) and SFA (saturated fatty acids) content, as well as the PUFA/SFA ratio (Table 7). The PUFA content in the seeds ranged from 54.13% to 67.46%, with the ‘Mazrone’ variety identified as the grape variety with the highest PUFA content and the highest PUFA/SFA ratio (5.44). When the varieties were classified according to their intended use, it was observed that table grape varieties had a higher SFA content than wine and raisin varieties (*p* < 0.05).

In previous studies, grape seeds have also been ranked in the order PUFA > MUFA > SFA according to the saturation status of their fatty acids [77,78]. Furthermore, many researchers have reported that grape varieties differ from one another in terms of the SFA, MUFA and PUFA content of their seed oil [11,14,79]. Various studies have demonstrated that the PUFA/SFA ratio in seed oils, both in grapevines and in certain other fruit species, can be influenced by irrigation, fertilisation and other agricultural management practices [70,80,81,82]. In contrast to previous studies, our research—which examined the composition of seed oils from samples of the Çirbeyt variety collected from different vineyards within the same ecological region, taking into account their degree of saturation—suggests that the effects of cultural practices applied to the varieties on fatty acids are quite limited. Based on this, it can be suggested that the fatty acid composition of seed oil should be used in addition to other ampelographic indicators for the identification and comparison of genotypes belonging to the *Vitis vinifera* L. species that occur naturally within the same ecological region.

**Figure 6:**
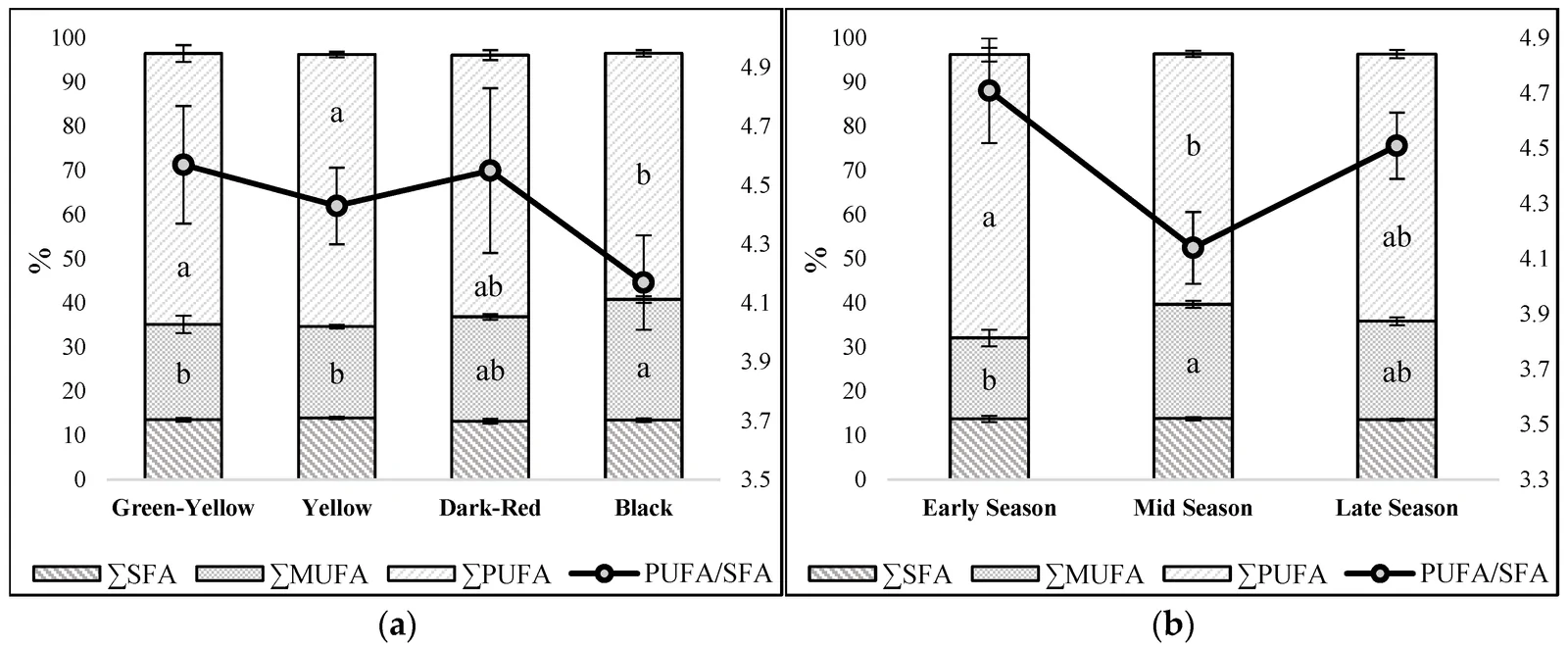
Variation in fatty acid saturation in grape seeds across berry skin colour (**a**) and ripening stage (**b**) groups. Different lowercase letters indicate statistically significant differences among groups according to Tukey’s multiple comparison test.

The PUFA/SFA ratio was above 3.97 in all varieties. This relatively high ratio indicates a fatty acid profile that may be considered nutritionally favourable in the context of current dietary recommendations favouring the replacement of saturated fatty acids with polyunsaturated fatty acids [83,84]. In a comparison based on skin colour groups, it was found that black-skinned varieties had a higher MUFA content (*p* < 0.05) and a lower PUFA content (*p* < 0.01) (Figure 6a). With regard to the ripening period, it was found that early-maturing varieties had a significantly higher PUFA ratio (*p* < 0.01) and a lower MUFA content (*p* < 0.05) (Figure 6b). No statistically significant differences were found in the PUFA/SFA ratio between either the husk colour or the seed maturity stage groups (*p* > 0.05).

### 2.6. Correlation

**Figure 7:**
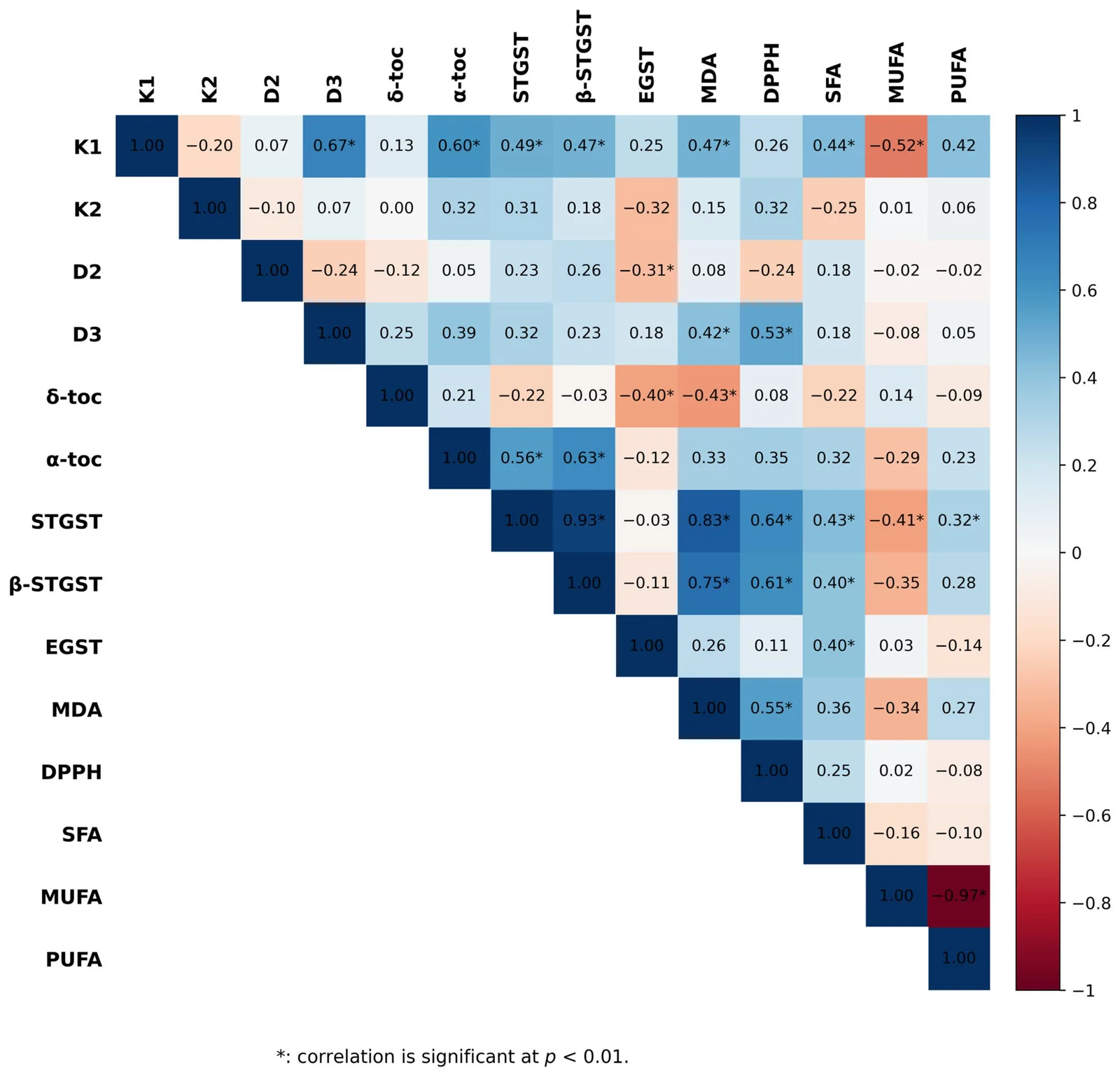
Pearson’s correlation coefficients of phytochemical compounds found in grape seeds.

A correlation plot illustrating the interrelationships between the amounts of the phytochemicals analysed in the seeds of different grape varieties—regardless of variety—is presented in Figure 7. According to this, the strongest negative correlation (r = −0.97, *p* < 0.01) was observed between MUFA and PUFA. This is due to the inverse relationship between linoleic acid and oleic acid. Studies examining different grape varieties have also frequently highlighted the relationship between these two fatty acids [61,73,85]. Furthermore, in parallel with the increase in MUFA content in the seeds, the levels of vitamin K_1_, stigmasterol, β-sitosterol and MDA decreased. In parallel with the increase in SFA, the levels of vitamin K_1_, α-tocopherol, sterols and MDA in the seeds increased.

It has been found that, in grape seeds—which possess high antioxidant capacity—levels of α-tocopherol, stigmasterol, β-sitosterol, MDA and vitamins K_2_ and D_3_ also increase in parallel with the rise in antioxidant capacity (DPPH). Zhao et al. [66], who reached similar findings, reported a positive correlation between vitamin E content and DPPH in grape seeds. No relationship was detected between DPPH and fatty acid saturation groups. This finding has been reported by Yi et al. [10] in grape juice and by Çakır et al. [19] in grape seeds.

When the variations in vitamins within the seeds were examined individually, a statistically significant relationship (r = 0.67, *p* < 0.01) was observed only between K_1_ and D_3_. No significant (*p* > 0.05) relationship was found between the tocopherol groups. Although Sabir et al. [61] detected a positive correlation between α-tocopherol and δ-tocopherol in grape seeds, Çakır et al. [19] were unable to find this relationship in the varieties they examined. The strongest positive correlation (r = 0.93, *p* < 0.01) we observed in grape seeds was between stigmasterol and β-sitosterol. In contrast, the amount of ergosterol in the seeds was not affected by changes in the other two sterol compounds. Whilst the variation in phytochemicals in grape seeds may be influenced by numerous environmental factors, it is primarily shaped under the control of genotype. Therefore, the minor differences observed between studies in the literature, including our own, stem from these factors.

### 2.7. Principal Component Analysis (PCA)

**Figure 8:**
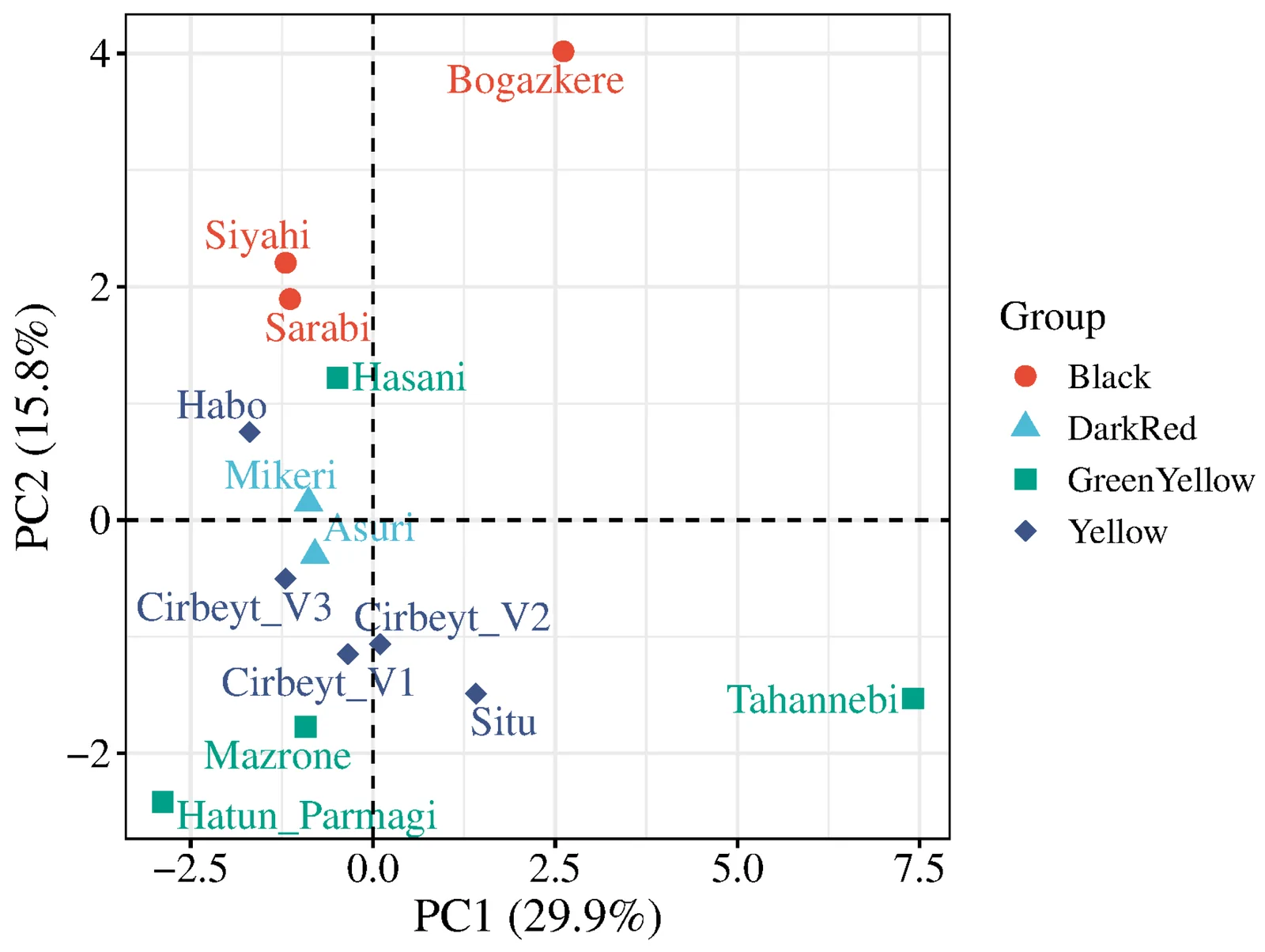
Principal component analysis (PCA) score plot of grape varieties based on standardized phytochemical variables measured in seeds. PC1 and PC2 explained 29.9% and 15.8% of the total variance, respectively. Samples were coloured according to berry skin colour groups.

Principal component analysis (PCA) was performed to obtain an integrated multivariate evaluation of the phytochemical composition of grape seed samples. The first two principal components explained 45.7% of the total variation, with PC1 and PC2 accounting for 29.9% and 15.8%, respectively (Figure 8). The PCA score plot revealed a partial grouping of samples according to berry skin colour; however, the overlap among groups indicated that skin colour alone did not fully explain the observed variation. Tahannebi was clearly separated along the positive side of PC1, whereas Boğazkere was distinguished mainly along PC2. In contrast, the Çirbeyt samples (*V1*, *V2* and *V3*) were positioned relatively close to each other, suggesting a similar phytochemical profile despite being collected from different vineyards. Overall, the PCA results supported the general conclusion of the study that varietal/genotypic differences played a more important role than berry skin colour in determining grape seed phytochemical composition.

## 3. Materials and Methods

### 3.1. Plant Material and Study Area

The research material consisted of seeds from twelve local grape (*Vitis vinifera* L.) varieties of economic importance in the regional viticulture of Eğil district, Diyarbakır province, Türkiye. The varieties were grown on their own roots and sampled from commercial vineyards located within the villages of Kazanlı, Selmanköy, Koçerli, Işıklı, and Konak. Ampelographic descriptions of the varieties were established in 2016, and local evaluation forms were prepared accordingly. Selected ampelographic characteristics of the examined varieties are presented in Table 8. Within the scope of the study, samples of the ‘Çirbeyt’ cultivar were collected from three different vineyards (*V1*, *V2*, and *V3*) and evaluated separately.

All grape varieties included in this study were cultivated in vineyards managed according to standard regional commercial practices. Vines were grown under rainfed conditions without supplemental irrigation, trained using the traditional goblet system, and spaced at 4 × 4 m. No mulching was applied either within or between rows, and annual winter pruning was performed. However, soil tillage, pest management, and fertilization practices were determined by individual growers.

The study area has a continental climate; in 2017, when the samples were taken, rainfall levels were lower (332 mm) than the long-term average (461 mm), although monthly average temperatures remained within seasonal norms [86]. For the contextual characterization of the study area, previously published soil data were used. According to Çiçek [87], the soils of the region are alkaline (pH 7.75), with an average organic matter content of 1.01% and lime content of 7.07%. The soil texture is clay loam (saturation: 66.8%), and the salinity level is low.

### 3.2. Sample Preparation

During the 2017 growing season, grapes from the varieties marked the previous year were harvested when their total soluble dry matter content reached the 18–21 Brix range. The seeds, removed from the berries using a scalpel, were washed with distilled water, dried at 40 °C for 3 h and subsequently ground in an agate mortar. The powdered samples were transferred to sealed tubes and stored at −80 °C only temporarily during laboratory preparation and procurement of the required analytical materials. The storage period did not exceed 15 days, and all laboratory analyses reported in the present study were completed during the 2017 experimental period.

For each grape variety/sample identity, three independent biological samples were collected and processed separately (*n* = 3). The three ‘Çirbeyt’ samples originating from different vineyards (*V1*, *V2*, and *V3*) were considered separate sample identities, each represented by three biological replicates.

### 3.3. Vitamin and Phytosterol Analyses

All chemicals and solvents used in the analyses were of analytical or HPLC grade and were purchased from Sigma-Aldrich (St. Louis, MO, USA).

Vitamin (K_1_, K_2_, D_2_ and D_3_) and phytosterol (β-sitosterol, stigmasterol and ergosterol) analyses were performed in 2017 using an HPLC-UV method. For this purpose, 1 g of seed sample was homogenised in a hexane/isopropanol mixture (3:2, *v*/*v*) and subsequently hydrolysed with 5% KOH at 85 °C. Unsaponifiable lipophilic compounds were extracted twice with 5 mL hexane. After evaporation of the hexane phase under a nitrogen stream, the residue was dissolved in 1 mL acetonitrile/methanol (3:2, *v*/*v*) and transferred to autosampler vials. Chromatographic analysis was performed using a Shimadzu LC-10 ADVP system equipped with an SPD-10AVP UV–Vis detector (Shimadzu Corporation, Kyoto, Japan) and a SUPELCOSIL™ LC-18 column (15 cm × 4.6 mm, 5 µm; Supelco, Bellefonte, PA, USA). Acetonitrile/methanol (3:2, *v*/*v*) was used as the mobile phase at a flow rate of 1.0 mL min^−1^. Detection was performed at 202 nm for α-tocopherol and phytosterols and at 265 nm for vitamins D and K. A comparable HPLC-UV analytical procedure was subsequently reported by Çakır et al. [19].

### 3.4. Determination of MDA Content

MDA content, an indicator of lipid peroxidation, was determined according to the method of Heath and Packer [88]. A 0.5 g seed sample was homogenised in 0.1% trichloroacetic acid (TCA) and centrifuged at 10,000 rpm. To the resulting 2 mL of supernatant, 2 mL of a 0.5% thiobarbituric acid (TBA, prepared in 20% TCA) solution was added, and the mixture was incubated at 95 °C for 30 min. The absorbance of the samples, which were cooled in an ice bath and centrifuged again, was measured at 532 and 600 nm. The MDA concentration was calculated using a molar absorption coefficient of 155 mM^−1^ cm^−1^ and the results were expressed in nmol g^−1^.

### 3.5. Determination of DPPH Radical Scavenging Capacity

The free radical scavenging capacity of grape seeds was determined according to the method developed by Brand-Williams et al. [20]. A 4 mL aliquot of a DPPH solution prepared in methanol at a concentration of 25 mg/L was mixed with plant extracts in varying volumes (50–800 µL) and incubated in the dark for 30 min. At the end of the incubation, the absorbance was measured at 517 nm and the percentage of inhibition was determined using the following formula (Equation (1)).

DPPH Inhibition (%) = [(Control ABS − Sample ABS)/Control ABS] × 100(1)

### 3.6. Determination of Fatty Acid Composition

Lipid extraction was carried out using a hexane/isopropanol (3:2, *v*/*v*) mixture, in accordance with the method of Hara and Radin [89]. The resulting lipid fraction was methylated with 2% methanolic sulfuric acid at 50 °C for 15 h. After cooling to room temperature, 5% sodium chloride solution was added, and the fatty acid methyl esters (FAMEs) were extracted with n-hexane. The hexane phase was treated with 2% KHCO_3_ solution and allowed to stand for phase separation. Subsequently, the hexane phase was evaporated under a nitrogen stream at 45 °C, and the FAME residues were dissolved in n-hexane and transferred to vials for analysis.

FAMEs were analyzed using a Shimadzu GC-17 gas chromatograph (Shimadzu Corporation, Kyoto, Japan) equipped with an SP™-2380 capillary column (30 m × 0.25 mm i.d., 0.20 µm film thickness; Supelco, Bellefonte, PA, USA). The injector and detector temperatures were maintained at 240 and 280 °C, respectively. The oven temperature was initially set at 120 °C and increased to 200 °C at 5 °C min^−1^, followed by an increase from 200 to 220 °C at 4 °C min^−1^. Nitrogen was used as the carrier gas. Individual FAMEs were identified by comparing their retention times with those of standard fatty acid methyl esters analyzed under the same chromatographic conditions. Quantification was performed using Class GC 10 software (Shimadzu Corporation, Kyoto, Japan) and the external standard method.

The relative proportion of each fatty acid was calculated as a percentage of the total identified fatty acids according to the following equation (Equation (2)):

Fatty acid (%) = [individual fatty acid content/total content of identified fatty acids] × 100.(2)

### 3.7. Statistical Analysis

Statistical analyses were based on three independent biological replicates for each of the 14 sample identities (n = 3 per sample identity; total N = 42 biological observations). Technical replicate measurements, where applicable, were not considered independent experimental units. One-way analysis of variance (one-way ANOVA) was applied to determine differences among sample identities, and Tukey’s multiple comparison test was used to compare means. The analyses were performed using IBM SPSS Statistics, version 20.0 (IBM Corp., Armonk, NY, USA).

The relationships among the measured variables were assessed using Pearson’s correlation analysis. Correlation coefficients (r) and corresponding significance levels (*p*) were calculated, and the resulting correlation matrix was visualized as a heatmap to facilitate the interpretation of relationships among variables.

In addition, principal component analysis (PCA) was performed using standardized (z-score-transformed) phytochemical variables for the 14 sample identities to evaluate the overall multivariate structure of the dataset and to visualize sample grouping patterns. Berry skin colour, ripening stage, and utilization type are cultivar-associated characteristics and are not fully orthogonal to varietal identity. Therefore, these categorical variables were not interpreted as independent experimental factors, and separate one-way comparisons were used only as exploratory group-wise analyses.

## 4. Conclusions

This study demonstrated substantial varietal variation in the phytochemical composition of grape seeds from indigenous *Vitis vinifera* L. cultivars grown in Southeastern Anatolia. Linoleic acid was the predominant fatty acid in all cultivars, while α-tocopherol and β-sitosterol were the major tocopherol and phytosterol components, respectively. The results further showed that ripening period and berry skin colour influenced selected phytochemical traits, whereas differences associated with utilization type were comparatively limited. Overall, these findings indicate that varietal characteristics represent a major source of variation in grape seed biochemical composition.

Among the evaluated cultivars, Tahannebi was distinguished by high levels of several bioactive constituents, whereas Mazrone exhibited the highest PUFA/SFA ratio. These results highlight the potential of local grape germplasm as a source of value-added compounds for food, nutraceutical, cosmetic, and related applications. In addition, the marked differences observed among cultivars suggest that the selection of grape varieties for industrial use should consider not only yield and technological characteristics but also seed phytochemical composition.

Nevertheless, the observed differences should be interpreted within the ecological and agronomic conditions of the present study. Future multi-year and multi-location studies involving grape varieties from different viticultural regions are warranted to validate the stability of these phytochemical profiles and to further elucidate genotype × environment interactions. Such studies may also help identify cultivars with consistently high-value biochemical profiles for targeted industrial applications.

## Figures and Tables

**Table 1:** Vitamin K and D contents in the seeds of grape varieties. Data are shown as means ± SEM.

Variety	K_1_ (µg g^−1^)	K_2_ (µg g^−1^)	D_2_ (µg g^−1^)	D_3_ (µg g^−1^)
Boğazkere	0.503 ± 0.009 d *	0.000 ± 0.000 f *	0.000 ± 0.000 j *	0.503 ± 0.009 b *
Siyahi	0.143 ± 0.003 l	0.000 ± 0.000 f	0.460 ± 0.006 d	0.053 ± 0.003 h
Şarabi	0.157 ± 0.003 kl	0.087 ± 0.003 e	0.127 ± 0.003 h	0.187 ± 0.003 f
Tahannebi	0.653 ± 0.003 b	0.253 ± 0.009 b	0.683 ± 0.007 c	0.377 ± 0.003 c
Hasani	0.207 ± 0.003 hi	0.000 ± 0.000 f	0.033 ± 0.003 i	0.283 ± 0.003 d
Hatun Parmağı	0.107 ± 0.003 m	0.387 ± 0.003 a	0.153 ± 0.003 g	0.357 ± 0.003 c
Mazrone	0.213 ± 0.003 h	0.157 ± 0.003 c	0.000 ± 0.000 j	0.157 ± 0.003 g
Çirbeyt (*V1*)	0.253 ± 0.003 g	0.000 ± 0.000 f	0.833 ± 0.009 b	0.153 ± 0.003 g
Çirbeyt (*V2*)	0.533 ± 0.003 c	0.000 ± 0.000 f	0.000 ± 0.000 j	0.577 ± 0.003 a
Çirbeyt (*V3*)	0.183 ± 0.003 ij	0.000 ± 0.000 f	0.403 ± 0.003 e	0.000 ± 0.000 i
Şitu	0.833 ± 0.003 a	0.000 ± 0.000 f	0.000 ± 0.000 j	0.377 ± 0.003 c
Habo	0.063 ± 0.003 n	0.127 ± 0.003 d	0.110 ± 0.006 h	0.053 ± 0.003 h
Mikeri	0.283 ± 0.003 f	0.153 ± 0.003 c	0.307 ± 0.003 f	0.203 ± 0.003 f
Asuri	0.437 ± 0.009 e	0.013 ± 0.003 f	1.603 ± 0.003 a	0.233 ± 0.009 e
Utilization Type				
Table	0.383 ± 0.069 ^Ns^	0.130 ± 0.036 ^Ns^	0.431 ± 0.139 ^Ns^	0.280 ± 0.028 ^Ns^
Wine	0.307 ± 0.037	0.041 ± 0.015	0.227 ± 0.074	0.263 ± 0.050
Raisin	0.213 ± 0.031	0.077 ± 0.034	0.383 ± 0.034	0.128 ± 0.034

There are statistically significant differences between the mean values indicated by different letters (*: *p* < 0.01). Ns: non-significant.

**Table 2:** Tocopherol contents in grape seeds. Data are shown as means ± SEM.

Variety	δ-Tocopherol (mg kg^−1^)	α-Tocopherol (mg kg^−1^)
Boğazkere	0.030 ± 0.006 i *	2.153 ± 0.009 L *
Siyahi	0.577 ± 0.009 d	1.597 ± 0.003 o
Şarabi	0.640 ± 0.006 c	2.643 ± 0.003 g
Tahannebi	0.360 ± 0.006 g	4.653 ± 0.015 b
Hasani	0.463 ± 0.003 e	3.403 ± 0.003 d
Hatun Parmağı	0.710 ± 0.006 b	3.557 ± 0.003 c
Mazrone	0.357 ± 0.003 g	2.357 ± 0.009 i
Çirbeyt (*V1*)	0.543 ± 0.009 d	2.740 ± 0.006 f
Çirbeyt (*V2*)	1.027 ± 0.003 a	2.237 ± 0.003 j
Çirbeyt (*V3*)	0.407 ± 0.007 f	2.023 ± 0.007 m
Şitu	0.733 ± 0.003 b	4.703 ± 0.003 a
Habo	0.320 ± 0.006 h	1.887 ± 0.003 n
Mikeri	0.477 ± 0.003 e	2.420 ± 0.006 h
Asuri	0.443 ± 0.009 e	2.757 ± 0.003 e
Utilization Type		
Table	0.505 ± 0.039 ^Ns^	3.493 ± 0.241 ^Ns^
Wine	0.501 ± 0.073	2.359 ± 0.062
Raisin	0.527 ± 0.023	2.342 ± 0.035

There are statistically significant differences between the mean values indicated by different letters (*: *p* < 0.01). Ns: non-significant.

**Table 3:** Phytosterol contents in the seeds of grape varieties. Data are shown as means ± SEM.

Variety	Stigmasterol (µg g^−1^)	β-Sitosterol (µg g^−1^)	Ergosterol (µg g^−1^)
Boğazkere	873.33 ± 0.507 b *	1359.00 ± 0.144 g *	385.17 ± 0.300 a *
Siyahi	716.50 ± 0.144 c	1779.75 ± 0.144 b	121.92 ± 8.168 c
Şarabi	491.17 ± 0.083 l	1472.17 ± 0.083 e	7.17 ± 0.083 gh
Tahannebi	2394.58 ± 0.083 a	3340.42 ± 0.300 a	9.42 ± 0.220 fgh
Hasani	558.50 ± 0.144 h	1401.08 ± 0.083 f	16.92 ± 0.083 fg
Hatun Parmağı	582.58 ± 0.083 g	1253.42 ± 0.083 j	21.92 ± 0.083 f
Mazrone	352.67 ± 0.083 n	915.67 ± 0.083 n	0.00 ± 0.000 h
Çirbeyt (*V1*)	502.75 ± 0.144 k	1640.33 ± 0.220 c	110.25 ± 0.144 c
Çirbeyt (*V2*)	602.08 ± 0.083 f	1351.42 ± 0.083 h	40.08 ± 0.083 d
Çirbeyt (*V3*)	522.75 ± 0.144 j	1296.33 ± 0.083 i	38.50 ± 0.144 d
Şitu	626.42 ± 0.083 e	1573.92 ± 0.083 d	238.75 ± 0.144 b
Habo	527.83 ± 0.220 i	957.67 ± 0.083 m	250.67 ± 0.083 b
Mikeri	408.33 ± 0.083 m	1058.83 ± 0.083 l	22.58 ± 0.083 f
Asuri	650.33 ± 0.220 d	1243.42 ± 0.083 k	1.25 ± 0.144 h
Utilization Type			
Table	890.04 ±163.49 ^Ns^	1628.32 ± 191.08 ^Ns^	89.82 ± 26.62 ^Ns^
Wine	557.46 ± 38.64	1339.15 ± 53.33	96.86 ± 32.44
Raisin	562.42 ± 68.91	1419.29 ± 161.20	72.25 ± 22.51

There are statistically significant differences between the mean values indicated by different letters (*: *p* < 0.01). Ns: non-significant.

**Table 4:** MDA content and antioxidant capacity (DPPH) in the seeds of grape varieties. Data are shown as means ± SEM.

Variety	MDA (nmol g^−1^)	DPPH (%)
Boğazkere	248.33 ± 0.33 b *	94.880 ± 0.003 d *
Siyahi	52.02 ± 0.01 f	95.067 ± 0.007 c
Şarabi	110.02 ± 0.01 d	93.600 ± 0.003 h
Tahannebi	314.50 ± 0.01 a	96.890 ± 0.003 a
Hasani	34.51 ± 0.01 l	94.702 ± 0.007 e
Hatun Parmağı	40.51 ± 0.01 k	94.515 ± 0.006 f
Mazrone	49.51 ± 0.01 g	92.505 ± 0.003 k
Çirbeyt (*V1*)	112.84 ± 0.67 c	93.235 ± 0.003 j
Çirbeyt (*V2*)	59.02 ± 0.01 e	95.245 ± 0.003 b
Çirbeyt (*V3*)	43.01 ± 0.01 j	91.412 ± 0.007 m
Şitu	58.51 ± 0.01 e	93.422 ± 0.002 i
Habo	35.50 ± 0.01 l	93.967 ± 0.004 g
Mikeri	44.50 ± 0.01 i	94.517 ± 0.004 f
Asuri	46.02 ± 0.01 h	92.145 ± 0.010 l
Utilization Type		
Table	88.26 ± 24.62 ^Ns^	94.28 ± 0.35 ^Ns^
Wine	103.79 ± 17.05	93.48 ± 0.32
Raisin	48.26 ± 1.68	94.79 ± 0.13

There is a statistically significant difference between the mean values indicated by different letters (*: *p* < 0.01). Ns: non-significant.

**Table 5:** Major fatty acid composition of grape seed oils (%). Data are shown as means ± SEM.

Variety	Palmitic Acid (C16:0)	Stearic Acid (C18:0)	Oleic Acid (C18:1c (n-9))	Linoleic Acid (C18:2c (n-6))
Boğazkere	7.90 ± 0.06 bc *	6.28 ± 0.09 a *	22.84 ± 1.17 bcd *	56.96 ± 1.10 b–e *
Siyahi	8.56 ± 0.25 abc	4.84 ± 0.22 bcde	26.52 ± 0.57 ab	53.68 ± 1.15 e
Şarabi	7.47 ± 0.39 c	4.65 ± 0.28 b–f	26.08 ± 0.72 ab	55.18 ± 1.05 cde
Tahannebi	8.88 ± 0.16 abc	6.05 ± 0.11 ab	13.80 ± 0.92 g	66.73 ± 1.29 a
Hasani	9.17 ± 0.34 abc	4.84 ± 0.31 b–e	23.70 ± 0.40 abc	56.07 ± 1.17 b–e
Hatun Parmağı	8.43 ± 0.39 abc	3.82 ± 0.16 ef	27.32 ± 0.70 a	54.02 ± 1.15 de
Mazrone	8.89 ± 0.40 abc	3.38 ± 0.17 f	14.73 ± 0.63 fg	67.06 ± 1.16 a
Çirbeyt (*V1*)	8.76 ± 0.23 abc	5.35 ± 0.29 a–d	18.70 ± 0.48 def	61.32 ± 0.90 abc
Çirbeyt (*V2*)	8.17 ± 0.25 abc	5.08 ± 0.17 a–e	19.45 ± 0.68 cde	61.10 ± 1.09 abc
Çirbeyt (*V3*)	7.89 ± 0.35 bc	4.87 ± 0.34 a–e	18.45 ± 0.49 ef	62.76 ± 1.23 ab
Şitu	10.10 ± 0.37 a	4.55 ± 0.29 c–f	16.69 ± 0.66 efg	62.71 ± 1.19 ab
Habo	9.60 ± 0.38 ab	4.80 ± 0.18 b–e	20.54 ± 0.70 cde	58.27 ± 1.25 b–e
Mikeri	8.01 ± 0.36 bc	4.03 ± 0.10 def	20.29 ± 0.69 cde	60.78 ± 1.09 a–d
Asuri	8.42 ± 0.53 abc	5.60 ± 0.29 abc	22.83 ± 0.55 bcd	57.06 ± 1.18 b–e
Utilization Type				
Table	9.10 ± 0.20 a **	4.94 ± 0.19 ^Ns^	20.81 ± 1.11 ^Ns^	59.14 ± 1.12 ^Ns^
Wine	8.18 ± 0.16 b	4.93 ± 0.23	20.04 ± 0.91	60.73 ± 1.01
Raisin	8.28 ± 0.23 b	4.43 ± 0.21	23.40 ± 1.45	57.23 ± 1.74

There are statistically significant differences between the mean values indicated by different letters (*: *p* < 0.01; **: *p* < 0.05). Ns: non-significant.

**Table 6:** Composition of minor and essential fatty acids in grape seed oils (%) Data are shown as means ± SEM.

Variety	Palmitoleic Acid (C16:1)	Trans-Elaidic Acid (C18:1t (n-9))	Linolenic Acid (C18:3 (n-6))	Myristic Acid (C14:0)	Pentadecanoic Acid (C15:0)	α-Linolenic Acid (C18:3 (n-3))
Boğazkere	0.51 ± 0.02 ^Ns^	1.36 ± 0.21 abc *	0.37 ± 0.09 ^Ns^	0.20 ± 0.01 a *	0.08 ± 0.00 a *	0.09 ± 0.01 a *
Siyahi	0.70 ± 0.06	1.68 ± 0.06 ab	0.36 ± 0.01	0.17 ± 0.01 ab	0.06 ± 0.00 a	0.10 ± 0.00 a
Şarabi	0.63 ± 0.05	1.71 ± 0.23 ab	0.42 ± 0.12	0.16 ± 0.02 ab	0.05 ± 0.01 a	0.11 ± 0.02 a
Tahannebi	0.53 ± 0.08	0.00 ± 0.00 d	0.34 ± 0.02	0.18 ± 0.03 ab	0.07 ± 0.02 a	0.10 ± 0.01 a
Hasani	0.73 ± 0.06	1.43 ± 0.13 abc	0.41 ± 0.05	0.17 ± 0.03 ab	0.06 ± 0.01 a	0.10 ± 0.01 a
Hatun Parmağı	0.76 ± 0.07	1.84 ± 0.12 a	0.26 ± 0.03	0.03 ± 0.00 c	0.05 ± 0.01 a	0.00 ± 0.00 b
Mazrone	0.71 ± 0.06	0.85 ± 0.06 c	0.34 ± 0.02	0.16 ± 0.03 ab	0.05 ± 0.01 a	0.06 ± 0.01 ab
Çirbeyt (*V1*)	0.67 ± 0.07	1.16 ± 0.09 abc	0.27 ± 0.05	0.12 ± 0.01 abc	0.00 ± 0.00 b	0.08 ± 0.02 a
Çirbeyt (*V2*)	0.66 ± 0.10	1.24 ± 0.11 abc	0.25 ± 0.02	0.14 ± 0.01 abc	0.04 ± 0.01 ab	0.08 ± 0.01 a
Çirbeyt (*V3*)	0.60 ± 0.06	1.15 ± 0.03 abc	0.24 ± 0.01	0.14 ± 0.02 ab	0.03 ± 0.00 ab	0.08 ± 0.01 a
Şitu	0.76 ± 0.10	0.99 ± 0.14 bc	0.35 ± 0.08	0.16 ± 0.03 ab	0.06 ± 0.01 a	0.08 ± 0.02 a
Habo	0.88 ± 0.07	1.41 ± 0.13 abc	0.32 ± 0.03	0.17 ± 0.00 ab	0.06 ± 0.01 a	0.09 ± 0.01 a
Mikeri	0.68 ± 0.09	1.41 ± 0.11 abc	0.31 ± 0.06	0.23 ± 0.01 a	0.04 ± 0.01 ab	0.09 ± 0.01 a
Asuri	0.66 ± 0.09	1.43 ± 0.22 abc	0.27 ± 0.03	0.08 ± 0.01 bc	0.04 ± 0.01 ab	0.10 ± 0.01 a
Utilization Type						
Table	0.72 ± 0.04 ^Ns^	1.19 ± 0.15 ^Ns^	0.32 ± 0.02 ^Ns^	0.13 ± 0.02 b *	0.06 ± 0.00 ^Ns^	0.08 ± 0.01 ^Ns^
Wine	0.63 ± 0.03	1.24 ± 0.08	0.31 ± 0.03	0.15 ± 0.01 ab	0.04 ± 0.01	0.08 ± 0.01
Raisin	0.69 ± 0.05	1.55 ± 0.08	0.33 ± 0.03	0.20 ± 0.02 a	0.05 ± 0.01	0.10 ± 0.01
Skin Colour						
Green-Yellow	0.68 ± 0.04 ^Ns^	1.03 ± 0.21 ^Ns^	0.34 ± 0.02 ^Ns^	0.14 ± 0.02 ^Ns^	0.06 ± 0.00 ab **	0.06 ± 0.01 ^Ns^
Yellow	0.71 ± 0.04	1.19 ± 0.05	0.28 ± 0.02	0.15 ± 0.01	0.04 ± 0.01 b	0.08 ± 0.01
Dark-Red	0.67 ± 0.05	1.42 ± 0.11	0.29 ± 0.03	0.16 ± 0.03	0.04 ± 0.00 b	0.09 ± 0.01
Black	0.62 ± 0.04	1.58 ± 0.11	0.38 ± 0.04	0.18 ± 0.01	0.06 ± 0.01 a	0.10 ± 0.01
Ripening Season						
Early Season	0.60 ± 0.06 b **	0.71 ± 0.32 b *	0.32 ± 0.03 ^Ns^	0.21 ± 0.02 a *	0.05 ± 0.01 ^Ns^	0.09 ± 0.01 ^Ns^
Mid Season	0.76 ± 0.04 a	1.53 ± 0.08 a	0.32 ± 0.02	0.11 ± 0.02 b	0.05 ± 0.00	0.07 ± 0.01
Late Season	0.66 ± 0.03 ab	1.27 ± 0.07 a	0.32 ± 0.02	0.16 ± 0.01 ab	0.05 ± 0.01	0.08 ± 0.00

There are statistically significant differences between mean values denoted by different letters (*: *p* < 0.01; **: *p* < 0.05). Ns: non-significant.

**Table 7:** SFA, MUFA, and PUFA compositions and PUFA/SFA ratio in grape seed oils. Data are shown as means ± SEM.

Variety	∑SFA	∑MUFA	∑PUFA	PUFA/SFA
Boğazkere	14.46 ± 0.33 ab *	24.70 ± 0.88 c–f *	57.42 ± 1.00 cde *	3.97 ± 0.00 b–e *
Siyahi	13.63 ± 0.33 ab	28.90 ± 0.58 ab	54.13 ± 1.14 e	3.99 ± 0.00 e
Şarabi	12.33 ± 0.58 b	28.42 ± 0.58 abc	55.71 ± 1.19 de	4.56 ± 0.33 cde
Tahannebi	15.18 ± 0.33 a	14.33 ± 0.88 k	67.17 ± 1.26 ab	4.43 ± 0.33 b
Hasani	14.23 ± 0.58 ab	25.87 ± 0.58 a–d	56.59 ± 1.21 de	4.00 ± 0.00 b–e
Hatun Parmağı	12.33 ± 0.58 b	29.91 ± 0.58 a	54.28 ± 1.18 e	4.43 ± 0.33 de
Mazrone	12.48 ± 0.33 ab	16.29 ± 0.58 jk	67.46 ± 1.17 a	5.44 ± 0.33 a
Çirbeyt (*V1*)	14.24 ± 0.58 ab	20.52 ± 0.33 fgi	61.67 ± 0.97 a–d	4.35 ± 0.33 abc
Çirbeyt (*V2*)	13.43 ± 0.33 ab	21.35 ± 0.88 e–i	61.43 ± 1.10 a–d	4.59 ± 0.33 a-d
Çirbeyt (*V3*)	12.94 ± 0.58 ab	20.20 ± 0.58 gij	63.09 ± 1.25 abc	4.92 ± 0.58 ab
Şitu	14.87 ± 0.58 ab	18.45 ± 0.88 ijk	63.13 ± 1.25 abc	4.27 ± 0.33 ab
Habo	14.62 ± 0.58 ab	22.83 ± 0.58 d–g	58.67 ± 1.22 cde	4.03 ± 0.00 b–e
Mikeri	12.31 ± 0.58 b	22.38 ± 0.88 d–i	61.18 ± 1.15 b–d	4.99 ± 0.00 a–e
Asuri	14.14 ± 0.88 ab	24.92 ± 0.58 b–e	57.42 ± 1.22 cde	4.10 ± 0.33 b–e
Utilization Type				
Table	14.23 ± 0.30 a **	22.72 ± 1.25 ^Ns^	59.54 ± 1.13 ^Ns^	4.21 ± 0.10 ^Ns^
Wine	13.31 ± 0.27 ab	21.91 ± 0.95	61.13 ± 1.00	4.64 ± 0.15
Raisin	12.97 ± 0.42 b	25.64 ± 1.52	57.66 ± 1.73	4.49 ± 0.28

There are statistically significant differences between the mean values indicated by different letters (*: *p* < 0.01; **: *p* < 0.05). Ns: non-significant.

**Table 8:** Some ampelographic characteristics of the grape varieties examined.

Variety	Skin Colour	Utilization Type	Flower	Seed Count	Ripening Season
Tahannebi	Green-Yellow	Table	Pistillate	1-2	Early Season
Hasani	Green-Yellow	Table	Hermaphrodite	2-3	Mid Season
Hatun Parmağı	Green-Yellow	Table	Hermaphrodite	2-4	Mid Season
Mazrone	Green-Yellow	Wine-Must	Hermaphrodite	2-3	Late Season
Çirbeyt	Yellow	Wine-Must	Hermaphrodite	2-3	Late Season
Şitu	Yellow	Table	Hermaphrodite	2-3	Late Season
Habo	Yellow	Table	Hermaphrodite	1-3	Mid Season
Mikeri	Dark Red-Purple	Raisin	Hermaphrodite	2-3	Early Season
Asuri	Dark Red-Black	Table	Hermaphrodite	2-3	Mid Season
Boğazkere	Black	Wine-Must	Hermaphrodite	2-3	Late Season
Siyahi	Black	Raisin	Hermaphrodite	2-3	Late Season
Şarabi	Black	Wine-Must	Hermaphrodite	2-3	Late Season

## Data Availability

The data presented in this study are available from the corresponding author upon reasonable request.
